# Predictors of workplace absenteeism in cancer care workers

**DOI:** 10.3747/co.v16i6.427

**Published:** 2009-12

**Authors:** A.J. Carosi, N.E. Lightfoot

**Affiliations:** * Regional Cancer Program, Sudbury Regional Hospital, Sudbury, ON

**Keywords:** Absenteeism, cancer care workers

## 1. INTRODUCTION

Workplace absenteeism occurs at an increased rate among employees in health care as compared with employees in other sectors 1. In the general workplace, absenteeism has been shown to vary with demographics and occupational characteristics 1–10. The Regional Cancer Program of the Sudbury Regional Hospital conducted a retrospective review of evidence in cancer care workers for factors that may predict a high frequency of sickness absenteeism in the workplace.

## 2. METHODS

Attendance records were used to collect data on all employee paid and unpaid sick leave. Factors examined included age, sex, occupational title, job level, employment contract (permanent or temporary), employment status (full- or part-time), duration of employment, and subprogram (clinical, nonclinical, administration).

## 3. RESULTS

Our study included 244 (95%) of all cancer care workers who were eligible for benefits between January 1, 1998, and December 31, 2003. Twelve employees were excluded from the study: 10 employees who refused to participate, and 2 employees with less than 12 months’ cumulative work experience.

In a univariate analysis, age, sex, job level, and duration of employment were significantly associated with high sickness absenteeism (Table I). Full-time workers were more likely to demonstrate a high frequency of sickness absence than were temporary workers; permanent workers were more likely to be absent than were temporary workers; and clinical and administrative employees were more likely to be absent more frequently than were nonclinical employees; however, these findings were not statistically significant.

In a logistic regression model (Table II), younger employees (less than 40 years of age) had a risk of high sickness absence that was significantly increased compared with the risk for older employees [odds ratio (or): 2.60; 95% confidence interval (ci): 1.44 to 4.72]. A significantly increased risk of high sickness absence (or: 2.08; 95% ci: 1.02 to 4.27) was also observed for low job level workers as compared with high job level workers. As compared with than female workers, male workers showed a significantly decreased risk of high sickness absence (or: 0.35; 95% ci: 0.16 to 0.78). A significantly decreased risk of high sickness absence (or: 0.35; 95% ci: 0.16 to 0.78) was observed in part-time workers as compared with full-time workers. As compared with workers employed for 5 years or more, workers employed for less than 5 years had a significantly decreased risk of high sickness absence (or: 0.42; 95% ci: 0.16 to 0.80).

## 4. DISCUSSION AND CONCLUSIONS

The study findings suggest that demographic and occupational factors are valid and reliable predictors of high sickness absence in cancer care workers. To our knowledge, this is the first study to identify predictors of workplace absenteeism in cancer care workers.

Demographic and occupational factors have been shown to be associated with sickness absenteeism in health care populations 2,4,5. The standard for high sickness absence was drawn from comparable studies of sickness absenteeism; however, because of limited evidence in the population under consideration, the guideline was subsequently expanded to include studies of cancer care workers.

In contrast with published studies on sickness absenteeism 3,11, evidence in the present study suggests that employment contract and subprogram are not associated with high sickness absenteeism. Roelen *et al.* 3 associated lower rates of sickness absence in temporary workers as compared with permanent workers with lack of job security. We observed no difference for clinical or nonclinical workers as compared with administrative workers; however, literature comparisons are difficult because of the nature of occupational administrative workers; however, literature comparisons are difficult because of the nature of occupational groups used across various studies.

Ongoing studies are needed, which should include evaluation of the roles of additional factors (job strain, job satisfaction, decision authority, bullying, health status, physical activity, and so on) and which should incorporate both quantitative and qualitative methods that examine other key factors associated with sickness absenteeism.

## Figures and Tables

**TABLE I:** Univariate analyses of employee characteristics by sickness absence frequency

Employee characteristics (*n*=244)	Frequency of sickness absence^a^				*p* Value
	Low		High		
	(*n*)	(%)	(*n*)	(%)	
Age					
<40 years	51	37.5	85	62.5	0.010^b^
40+ years	59	54.6	49	45.4	
Sex					
Male	40	72.7	15	27.3	0.000^b^
Female	70	37.0	119	63.0	
Employment contract					
Permanent	83	43.5	108	56.5	0.353
Temporary	27	50.9	26	49.1	
Employment status					
Part-time	19	54.3	16	45.7	0.273
Full-time	91	43.5	118	56.5	
Job level^c^					
Low	69	37.5	115	62.5	0.000^b^
High	39	67.2	19	32.8	
Subprogram					
Clinical	62	42.5	84	57.5	0.483
Nonclinical	40	50.6	39	49.4	
Administration	8	42.1	11	57.9	
Duration of employment					
<5 years	51	54.8	42	45.2	0.018^b^
5+ years	59	39.1	92	60.9	

a Low = fewer than 3 absence events per working year; high = 3 or more absence events per working year.

b Significant results ( *p* < 0.05) by two-tailed Fisher exact or Pearson chi-square test.

c Based on a multi-competency scale on which the pay band was less than $60,000 for low job level workers and more than $60,000 for high job level workers.

**TABLE II:** Logistic regression analysis^a^ of employee characteristics by high frequency of sickness absence

Employee characteristics (*n*=244)	High frequency of sickness absence^b^	
	or	95% ci
Age		
<40 years	2.60	1.44 to 4.72
40+ years	1	
Sex		
Male	0.23	0.11 to 0.49
Female	1	
Employment status		
Part-time	0.35	0.16 to 0.78
Full-time	1	
Job level		
Low	2.08	1.02 to 4.27
High	1	
Duration of employment		
<5 years	0.42	0.16 to 0.80
5+ years	1	

a Variables not retained in the final model: “employment contract” and “subprogram.”

b Three or more absence events per working year.

or = odds ratio; 95% ci = 95% confidence interval.
